# Psychosocial Mediators of Web-Based Interventions for Promoting a Healthy Lifestyle Among Chinese College Students: Secondary Analysis of a Randomized Controlled Trial

**DOI:** 10.2196/37563

**Published:** 2022-09-07

**Authors:** Wei Liang, Yanping Duan, Yanping Wang, Sonia Lippke, Borui Shang, Zhihua Lin, Hagen Wulff, Julien Steven Baker

**Affiliations:** 1 Center for Health and Exercise Science Research Department of Sport, Physical Education and Health Hong Kong Baptist University Hong Kong China (Hong Kong); 2 Department of Sport, Physical Education and Health Faculty of Social Sciences Hong Kong Baptist University Hong Kong China (Hong Kong); 3 Jacobs University Bremen Bremen Germany; 4 Department of Social Sciences Hebei Sport University Shijiazhuang China; 5 Sport Section Wuhan University Wuhan China; 6 Institute of Exercise and Public Health University of Leipzig Leipzig Germany

**Keywords:** web-based intervention, physical activity, fruit and vegetable consumption, college students, psychosocial mediators, lifestyle, randomized controlled trial, RCT, mobile phone

## Abstract

**Background:**

Web-based multiple health behavior change (MHBC) interventions have demonstrated effectiveness in promoting physical activity (PA) and fruit and vegetable consumption (FVC) among Chinese college students. However, there is limited research examining their effects on promoting a healthy lifestyle (ie, adhering to both PA and FVC behavioral recommendations) among Chinese college students. In addition, the salient psychosocial mediators of successful MHBC interventions need to be researched.

**Objective:**

This study aims to examine the effectiveness of a previous 8-week web-based MHBC program for promoting a healthy lifestyle and enhancing the psychosocial determinants (intention, self-efficacy, planning, and social support) of behavior change among Chinese college students. Furthermore, the study aims to identify whether changes in these psychosocial determinants mediate intervention effectiveness on the immediate and sustained lifestyle changes.

**Methods:**

This was a secondary analysis for a 3-arm randomized controlled trial. Chinese college students (N=552) were randomly assigned to 1 of 3 groups: a PA-first group (4-week PA intervention followed by 4-week FVC intervention), an FVC-first group (4-week FVC intervention followed by 4-week PA intervention), and a placebo control group. The intervention content was designed based on the health action process approach model. Data for analyses were collected at baseline (T0), postintervention assessment (T1), and 12-week follow-up assessment (T2).

**Results:**

At baseline, 13.9% (77/552) of the participants maintained a healthy lifestyle. After 8 weeks, more (200/552, 36.2%) participants achieved a healthy lifestyle. PA-first and FVC-first groups were, respectively, 3.24 times and 5 times more likely to adopt a healthy lifestyle than the control group at T1. After 12 weeks, 35.5% (196/552) of the participants adopted a healthy lifestyle. Intervention groups were approximately 2.99 times (PA first) and 4.07 times (FVC first) more likely to adopt a healthy lifestyle than the control group at T2. Intervention effects favored both intervention groups in self-efficacy and planning for PA and in intention and planning for FVC compared with the control condition. In addition, changes in PA self-efficacy and FVC intention mediated intervention effectiveness on the immediate lifestyle change after 8 weeks. Changes in FVC intention were identified as a salient mediator for facilitating sustained lifestyle change after 12 weeks.

**Conclusions:**

This study provides empirical evidence for the effectiveness of an 8-week theory- and web-based MHBC intervention program on promoting a healthy lifestyle, self-efficacy and planning for PA, and intention and planning for FVC among Chinese college students. These research findings add new knowledge to the underlying psychosocial mechanisms of successful MHBC interventions. Overall, this study has considerable implications for future web-based MHBC research and practice in terms of addressing PA self-efficacy and FVC intention and helping students to adopt and maintain a healthy lifestyle independently of whether PA or FVC is addressed first.

**Trial Registration:**

ClinicalTrials.gov NCT03627949; https://clinicaltrials.gov/ct2/show/NCT03627949

## Introduction

### Background

As common health-protective behaviors, regular physical activity (PA) and adequate fruit and vegetable consumption (FVC) have been proposed as the cornerstones of healthy living [1-3]. These 2 health-protective behaviors constitute a typical pattern of healthy lifestyles, which play a dominant role in improving individuals’ overall health [3,4]. An overwhelming body of evidence has demonstrated the considerable impact that increasing PA and FVC could have not only on reducing the morbidity of noncommunicable diseases (eg, cardiovascular diseases, certain types of cancer, gastrointestinal diseases, and obesity) but also on improving mental well-being and quality of life [2,5].

To promote PA and FVC, widely acknowledged behavioral recommendations have been formulated, suggesting that adults aged 18 to 64 years should perform at least 150 minutes of moderate-intensity PA (or at least 75 minutes of vigorous-intensity PA or an equivalent combination of moderate-to-vigorous PA) per week and consume at least five servings (400 g) of fruit and vegetables each day [6,7]. Notwithstanding the recommended behavioral guidelines for PA and FVC, the prevalence of physical inactivity and insufficient intake of fruit and vegetables in the adult population is exceedingly high, especially among college students [8]. In western countries, 23% to 60% of college students do not meet the PA recommendations, whereas <30% comply with the FVC recommendations [8-11]. A similar situation exists in China, where >40% of Chinese college students do not perform the recommended weekly amount of PA, and more than half do not adhere to the recommendation of a minimum 5 servings daily of FVC [12,13]. Therefore, promoting health among college students has become a common challenge in many countries and has stimulated research interest among health psychologists and behavioral scientists [8].

Over the past 4 decades, an increasing number of psychosocial theories have been developed to describe, explain, and predict changes in health behaviors, such as the social cognitive theory [14], health belief model [15], protection motivation theory [16], theory of planned behavior [17], transtheoretical model [18], and the health action process approach (HAPA) [19]. These have been followed by a series of theory-based interventions that seem promising for changing specific health behavior. In particular, interventions based on the HAPA, which integrates the merits of the stage and continuum characteristics of contemporary psychosocial models, have demonstrated remarkable effects on promoting PA, healthy diet, and related health behaviors [20-22].

Although such interventions have achieved singular success, most of them focused only on a specific health behavior and addressed different behaviors as categorically separate entities [23]. As such, the interrelationships among different health behaviors have been artificially disconnected. However, in real life, individuals usually tend to have multidimensional patterns of health behaviors rather than 1 specific behavior in isolation [24]. A key property of these behaviors is that they typically coexist as behavioral clusters or bundles [23-25]. For instance, 1 risk behavior (eg, sedentary behavior) often occurs with other risk behaviors (eg, excessive intake of fat and sugar, smoking, excessive sedentary screen time, or alcohol addiction), or 1 health-protective behavior coexists with other health-protective behaviors (eg, PA and FVC). This high co-occurrence of different health behaviors can generate synergistic or additive effects so that when promoting multiple health-protective behaviors simultaneously, the overall health benefits can be greatly increased [23-27]. As a result, interventions targeting multiple health behavior change (MHBC) have grown in popularity over the past decade as a potential and pragmatic way to maximize overall health outcomes. With the burgeoning use of internet technology, web-based MHBC interventions have been increasingly applied to a wide range of populations [8,28]. Compared with traditional face-to-face hand-delivered interventions this new paradigm has been praised for its numerous advantages, such as accessibility, scalability, cost-effectiveness, flexibility, and convenience [29].

One critical issue that remains understudied concerns the psychosocial mechanisms behind MHBC (ie, salient mediators of successful MHBC interventions) [24,27]. Psychosocial theory-based MHBC interventions have been advocated by many researchers as scientific theories that can provide a useful framework for addressing the key modifiable determinants (eg, motivation and volition) of health behavior that may consequently increase the effectiveness of MHBC interventions [24,27,30,31]. However, many so-called theory-based health interventions are probably better categorized as *theory inspired* rather than *theory based* because they may not apply the theory properly and extensively (eg, the intervention did not effectively link behavior change techniques to the theoretical components) [27,32]. As a result, considerable heterogeneity in the effect sizes is found in theory-based health interventions, and some reviews have even concluded that the use of theories has no bearing on the effectiveness of interventions [27,30-33]. Therefore, it is important to identify active mediators of intervention effectiveness and increase our understanding of theoretical constructs in terms of the magnitude of their impacts in different behavior interventions targeting different populations.

### Our Previous Web-Based MHBC Intervention Program for Chinese College Students

Given the urgent need for, and limited practice of, MHBC interventions for promoting both PA and FVC among Chinese college students, we developed an 8-week web-based MHBC intervention program. To address the debatable question in MHBC research (ie, how to deliver MHBC interventions to achieve more robust treatment effects or whether the order of the sequential intervention contents makes a difference) [34], we designed two sequentially delivered modules (ie, PA first and FVC first) in our previous program and compared the differences in their intervention effects on promoting PA and FVC among Chinese college students (details have been presented elsewhere [34]). Our previous program used the HAPA model as the theoretical backdrop [19]. The HAPA model postulates two distinctive phases of the behavior change process (ie, motivational and volitional phases), underlining the vital role of diverse psychosocial determinants in the behavior change process [35]. In the motivational phase, the primary task is to form a behavioral intention by reinforcing several crucial antecedents (eg, risk perception, outcome expectancies, and action self-efficacy). Once the behavioral intention has been formed, individuals need to enhance maintenance and recovery self-efficacies, apply a series of self-regulatory strategies (eg, action planning and coping planning), and use external resources (eg, social support) for facilitating the behavior initiation and maintenance in the volitional phase [36]. In our previous study, we evaluated the effectiveness of a web-based MHBC intervention program on promoting PA, FVC, and health-related outcomes (BMI, depression, and quality of life) among Chinese college students. The results supported favorable effects on both behaviors and BMI in the intervention groups compared with a control condition, with small-to-medium effect sizes (Cohen *d*=0.22-0.59), and indicated a superior effect on FVC maintenance in the FVC-first group compared with the PA-first group [34]. However, our previous analyses focused only on the change in each specific behavior, whereas the comparative intervention effects on the combination of multiple health behaviors (ie, lifestyle indicator) and psychosocial determinants of behavior change have not been examined. In addition, the underlying psychosocial mechanisms of successful MHBC (salient mediators) have not been identified.

### Objectives and Hypotheses

Given the aforementioned particulars, the first aim of this study was to examine the immediate and sustained effectiveness of our previous 8-week web-based intervention program for promoting a healthy lifestyle (ie, adhering to both PA and FVC behavioral recommendations) and enhancing the psychosocial determinants of PA and FVC (ie, intention, self-efficacy, planning, and social support) among Chinese college students. Furthermore, this study aimed to identify whether changes in psychosocial determinants of PA and FVC could account for the immediate and sustained lifestyle changes (ie, mediation analyses).

Correspondingly, the main intervention effects were hypothesized in terms of greater adoption of a healthy lifestyle (hypothesis 1) and more improvements in the psychosocial determinants of PA (hypothesis 2a) as well as FVC change (hypothesis 2b). The mediation effects were hypothesized in terms of the following assumption: participants in the intervention groups who had increased psychosocial determinants of PA and FVC would be more likely to have positive lifestyle changes after 8 (hypothesis 3a) and 12 weeks (hypothesis 3b) than those in the control group.

## Methods

### Ethics Approval

The study was approved by the research ethics committee of Hong Kong Baptist University (FRG2/15-16/032).

### Design, Participants, and Procedure

The study outlined herein has been described in greater detail elsewhere [34]. Data for the secondary analyses were collected in a 3-arm, double-blinded (ie, intervention facilitator and outcome evaluator) randomized controlled trial (RCT) evaluating sequentially delivered web-based interventions for PA and FVC among Chinese college students (ClinicalTrials.gov NCT03627949) [34].

In our previous RCT, the participants (N=634) were recruited from 28 different departments (the total number of departments is 34) of 1 university in the central region of China. The eligibility criteria were as follows: participants (1) were aged ≥18 years, (2) were not collegiate athletes or had not majored in any sport-related subjects, (3) had no contraindications to physical mobility (eg, cardiovascular diseases and disabilities) or FVC (fruit allergies or diabetes), and (4) had access to the internet and digital devices (eg, desktop computer, laptop computer, and smartphone). Details of the sampling approach, sample size estimate, recruitment procedure, and CONSORT (Consolidated Standards of Reporting Trials) flow diagram are described in detail in the primary paper [34].

After enrollment and eligibility checks, the eligible participants (N=556) were randomly assigned to 1 of 3 groups, which included a PA-first group (4 weeks of PA treatment followed by 4 weeks of FVC treatment), an FVC-first group (4 weeks of FVC treatment followed by 4 weeks of PA treatment), and a control group (8 weeks of placebo treatment irrelevant to either PA or FVC). The study was implemented from October 2017 to March 2018.

Of the 556 eligible participants, we excluded 4 (0.7%) who did not complete the baseline assessment; thus, the final sample considered for the analysis of this study consisted of 552 (99.3%) participants, specifically 187 (33.9%) in the PA-first group, 195 (35.3%) in the FVC-first group, and 170 (30.8%) in the control group. For the study analyses, measurements were recorded at baseline (T0), after the intervention (T1; 8 weeks after T0), and at follow-up (T2; 12 weeks after T0).

### Intervention

The intervention has been described in greater detail in our previously published papers [34-37]. The intervention content was designed based on the theoretical framework of the HAPA [19], lasting for 8 weeks with 1 session per week (each session lasting for between 20 and 30 minutes). Two sequentially delivered health interventions (ie, PA first and FVC first) were designed to target the HAPA-based psychosocial determinants of PA and FVC change. Considering that >90% of the Chinese college students were intenders and actors for PA and FVC behavior in our previous pilot study [38], this study focused more on the enhancement of intention, self-efficacy, planning, and social support to facilitate the crucial transition from intention to actual behavior initiation and maintenance (ie, intention-behavior gap).

In brief, for the PA-first group, the first 4-week intervention targeted the following psychosocial determinants of PA change:

- Week 1: risk perception, outcome expectancies, and goal settings (these antecedent variables contributed to the formation and enhancement of PA intention)
- Week 2: development of action planning
- Week 3: revision and adjustment of previous action planning and development of coping planning
- Week 4: revision and adjustment of previous coping planning and development of perceived social support

The same intervention materials were subsequently implemented to target the psychosocial determinants of FVC change in the second 4-week intervention period. For the FVC-first group, the sequence of intervention delivery was the reverse of the PA-first module. Self-efficacy was involved as a settled component throughout the entire intervention period. For the control condition, to avoid social desirability and the Hawthorne effect [39], all participants in the control group received active control treatments that seemed in all respects to be identical to the 2 intervention groups (eg, intervention duration, frequency, implementation procedure, and delivery modes) but lacked the critical psychosocial ingredients for changing PA or FVC [34].

The whole intervention, which consisted of three independent modules (ie, 2 MHBC intervention modules and 1 placebo control module), was delivered through a well-established platform. Participants were asked to attend the corresponding intervention session once a week through a laptop computer or desktop computer. WeChat (a popular social media platform in China) groups were established for participants who were included in the same intervention condition. Each participant received a WeChat group reminder that was distributed by the research team 1 day before the new intervention session [34].

### Measures

#### Lifestyle Indicator

The lifestyle indicator reflected the combination of multiple health behaviors (ie, whether the participant had complied with behavioral recommendations for both PA and FVC). We used the World Health Organization–recommended thresholds of at least 150 minutes of moderate-intensity PA per week (or at least 75 minutes of vigorous-intensity PA or an equivalent combination of moderate-to-vigorous PA) and 5 daily servings (400 g) of fruit and vegetables [6,7]. In our previous study, the weekly amount of PA was assessed using the Chinese brief version of the International Physical Activity Questionnaire [40], and the daily portion of FVC was evaluated using a Chinese version of the 4-item FVC scale [41]. Participants were categorized into 1 of 2 groups depending on whether they adhered to both PA and FVC recommendations (0=unhealthy lifestyle that met neither of the behavioral recommendations or only 1 behavioral recommendation and 1=healthy lifestyle that met both behavioral recommendations) [42,43].

#### Psychosocial Determinants of Behavior Change

##### Intention

Intention for PA was measured with the question stem “I intend to perform at least 30 minutes a day on minimum 5 days a week for at least 150 minutes per week with...” followed by 3 items: “...vigorous PA,” “...moderate PA,” and “...mild PA” (Cronbach α=.64). Intention for FVC was assessed by the question stem “I seriously intend to...” followed by 3 items: “...eat at least five servings of fruit and vegetables every day,” “...eat more fruit and vegetables each meal,” and “...drink at least one glass of fruit or vegetable juice every day” (Cronbach α=.63). The answers were indicated on a visual analog scale (VAS) ranging from *1*=*not true* to *4*=*exactly true* [34,38,43,44].

##### Self-efficacy

Self-efficacy was measured with the question stem “I am certain that...” followed by 5 items for PA such as “...I can be physically active on a permanent and regular basis (eg, at least 30 minutes a day on minimum 5 days a week), even if I have to overcome some barriers” or followed by 5 items for FVC such as “...I can eat 5 portions of fruit and vegetables a day even if it is sometimes difficult” (Cronbach α for PA=.88 and Cronbach α for FVC=.92). The answers were indicated on a VAS ranging from *1*=*don’t agree at all* to *5*=*agree completely* [34,38,43-45].

##### Planning

Planning includes two components: action planning and coping planning. Action planning was measured by the question stem “For the next month I already planned in detail...” followed by 3 items for PA such as “...which concrete PA I will pursue” or followed by 3 items for FVC such as “...how I will prepare the food” (Cronbach α for PA=.86 and Cronbach α for FVC=.91). Coping planning was measured by the question stem “For the next month I already planned in detail...” followed by 3 items for PA such as “...how I can stay active, even if something happened” or followed by 3 items for FVC such as “...what I can do in difficult situations, in order to remain true to my own resolutions” (Cronbach α for PA=.87 and Cronbach α for FVC=.93). Answers were given on a VAS ranging from *1*=*totally disagree* to *5*=*totally agree* [34,38,43-46].

##### Social Support

Perceived social support was measured by the question stem “How do you perceive your environment?” followed by 3 items for PA such as “People like my classmates and friends help me to stay physically active” or followed by 3 items for FVC such as “People like my classmates and friends help me to eat healthily” (Cronbach α for PA=.72 and Cronbach α for FVC=.69). Answers were given on a VAS ranging from *1*=*disagree* to *4*=*agree* [34,38,43-46].

##### Covariates

The covariates included age, sex, college grade (freshman, sophomore, junior, or senior), marital status (single or in a relationship), perceived health status (poor, satisfactory, or excellent), and BMI (kg/m^2^) [47].

All the questionnaires were written in simple Chinese and had been validated in previous studies using Chinese adult populations [34,38,43]. Sociodemographic information was collected only at registration, whereas all other indicators were assessed at baseline (T0), postintervention assessment (T1), and 12-week follow-up assessment (T2).

#### Statistical Analyses

Data analyses were performed using SPSS software (version 27.0; IBM Corp; eg, descriptive tests and intervention effect evaluation) and PROCESS macro (version 4.0; Andrew F Hayes; mediation analyses). Baseline characteristics and randomization were checked using independent 2-tailed *t* tests, ANOVA, and chi-square tests. Missing values were imputed using the multiple imputation approach with chained equations, except for dropouts, which were addressed using the baseline-observation-carried-forward approach [48]. The 5% level (2-tailed) was used as the statistical significance cutoff point.

With an intention-to-treat principle, intervention effects on the lifestyle indicator were examined using logistic regression analyses (determining odds ratios; hypothesis 1). For intervention effects on psychosocial determinants of behavior change (hypotheses 2a and 2b), generalized linear mixed models were used using a restricted maximum likelihood approach with time, group, and their interaction as fixed effects adjusted for the random effects of baseline behaviors. Unstructured covariance matrix was selected based on the minimal values of −2 log likelihood and Akaike and Bayesian information criteria. The least significant difference method was used for the post hoc comparison [49].

For hypotheses 3a and 3b, to control for the effects of baseline values, residualized change scores were used for the multiple mediation analyses [50]. The standardized coefficients and 95% CIs for direct, indirect, and total effects were estimated using the bias-corrected bootstrap approach (5000 resamples). The multicollinearity of psychosocial mediators was checked before the mediation analyses using the following criteria for an ignorable multicollinearity problem: low correlation (≤0.70), high tolerance (>0.01), low variance inflation factor (≤10), high eigenvalue (not approaching 0), and small condition index (≤30) [51]. For effect size *R*^2^, the proposed small, medium, and large values were 0.02, 0.13, and 0.26, respectively [52].

## Results

### Sample Characteristics and Randomization Check

A total of 552 participants (n=322, 58.3%, women) were included in the data analysis, with their ages ranging from 18 to 24 (mean 19.99, SD 1.04) years. Table 1 presents the descriptive information of the study sample in terms of their sociodemographic data, baseline values of psychosocial determinants for PA and FVC, and behavioral indicators at baseline.

Randomization checks indicated that there were no significant differences in baseline characteristics across the 3 groups in relation to age, sex, college grade, marital status, perceived health status, and BMI (*P*=.37-.83). In addition, the 3 groups did not vary significantly in all psychosocial mediators and behavioral indicators (*P*=.10-.93). Therefore, the randomization was successful.

At baseline (T0), 27.9% (154/552) of the participants did not meet the weekly PA recommendation, whereas 80.4% (444/552) did not consume at least five portions of fruit and vegetables per day. When both behaviors were combined, 86.1% (475/552) of the participants met only 1 or none of these 2 behavioral recommendations and were categorized as adopting unhealthy lifestyles at baseline. Overall, 13.9% (77/552) of the participants achieved both behavioral recommendations and were categorized as adopting healthy lifestyles.

At T1, 23.2% (128/552) of the participants did not meet the PA recommendation, whereas 55.6% (207/552) did not achieve the recommended daily servings of fruit and vegetables. When both behaviors were combined, 36.2% (200/552) of the participants met both behavioral recommendations and were categorized as having healthy lifestyles.

At T2, the percentage of participants adhering to the PA recommendation was 21.2% (117/552), whereas 43.8% (242/552) met the FVC recommendation. Taking both behaviors together, 35.5% (196/552) of the participants complied with both PA and FVC recommendations and were categorized as adopting healthy lifestyles.

**Table 1:** Sociodemographic information, psychosocial mediators, and behavioral indicators of the study sample at baseline.

Variable					Total (N=552)			PA^a^-first group (n=187)			FVC^b^-first group (n=195)			Control group (n=170)	
**Sociodemographic information**															
	Age (range 18-24 years), mean (SD)					19.99 (1.04)			20.07 (1.07)			19.96 (0.99)			19.93 (1.06)
	**Sex, n (%)**														
			Male			230 (41.7)			79 (42.2)			78 (40)			73 (42.9)
			Female			322 (58.3)			108 (57.8)			117 (60)			97 (57.1)
	**College grade, n (%)**														
			Freshman			264 (47.8)			86 (46)			90 (46.2)			88 (51.8)
			Sophomore			229 (41.5)			77 (41.2)			84 (43.1)			68 (40)
			Junior			46 (8.3)			18 (9.6)			16 (8.2)			12 (7.1)
			Senior			13 (2.4)			6 (3.2)			5 (2.6)			2 (1.2)
	**Marital status, n (%)**														
			Single		506 (91.7)			170 (90.9)			183 (93.8)			153 (90)	
			In a relationship		46 (8.3)			17 (9.1)			12 (6.2)			17 (10)	
	**Perceived health status, n (%)**														
			Poor		17 (3)			5 (2.7)			9 (4.6)			3 (1.8)	
			Satisfactory		358 (64.9)			122 (65.2)			125 (64.1)			111 (65.3)	
			Excellent		177 (32.1)			60 (32.1)			61 (31.3)			56 (32.9)	
	BMI (range 15.62-32.88 kg/m^2^), mean (SD)				20.41 (2.45)			20.32 (2.34)			20.52 (2.62)			20.40 (2.39)	
**Psychosocial determinants, mean (SD)**															
		PA intention		2.22 (0.71)			2.26 (0.74)			2.22 (0.72)			2.17 (0.69)		
		PA self-efficacy		2.96 (1.19)			3.08 (1.22)			2.87 (1.16)			2.92 (1.2)		
		PA planning		3.03 (1.05)			3.10 (0.98)			2.97 (1.04)			3.04 (1.12)		
		PA social support		2.23 (0.91)			2.25 (0.90)			2.24 (0.94)			2.19 (0.9)		
		FVC intention		1.96 (0.79)			1.93 (0.76)			1.97 (0.81)			1.99 (0.81)		
		FVC self-efficacy		3.08 (1.37)			3.07 (1.34)			3.06 (1.36)			3.11 (1.42)		
		FVC planning		2.86 (1.16)			2.83 (1.16)			2.83 (1.14)			2.94 (1.18)		
		FVC social support		2.37 (0.86)			2.40 (0.83)			2.38 (0.89)			2.32 (0.86)		
**Behavioral indicators, mean (SD)**															
		PA (minutes per week)		465.85 (257.29)			482.63 (269.42)			452.80 (248.94)			462.38 (253.51)		
		FVC (portions per day)		3.81 (1.75)			3.84 (1.70)			3.82 (1.87)			3.76 (1.68)		
**Lifestyle indicator, n (%)**															
		Unhealthy^c^		475 (86.1)			161 (86.1)			166 (85.1)			148 (87.1)		
		Healthy^d^		77 (13.9)			26 (13.9)			29 (14.9)			22 (12.9)		

^a^PA: physical activity.

^b^FVC: fruit and vegetable consumption.

^c^Participants adopted unhealthy lifestyles that met neither of the behavioral recommendations or only 1 behavioral recommendation.

^d^Participants adopted healthy lifestyles that met both behavioral recommendations.

### Intervention Effects on Lifestyle Indicator

**Figure 1:**
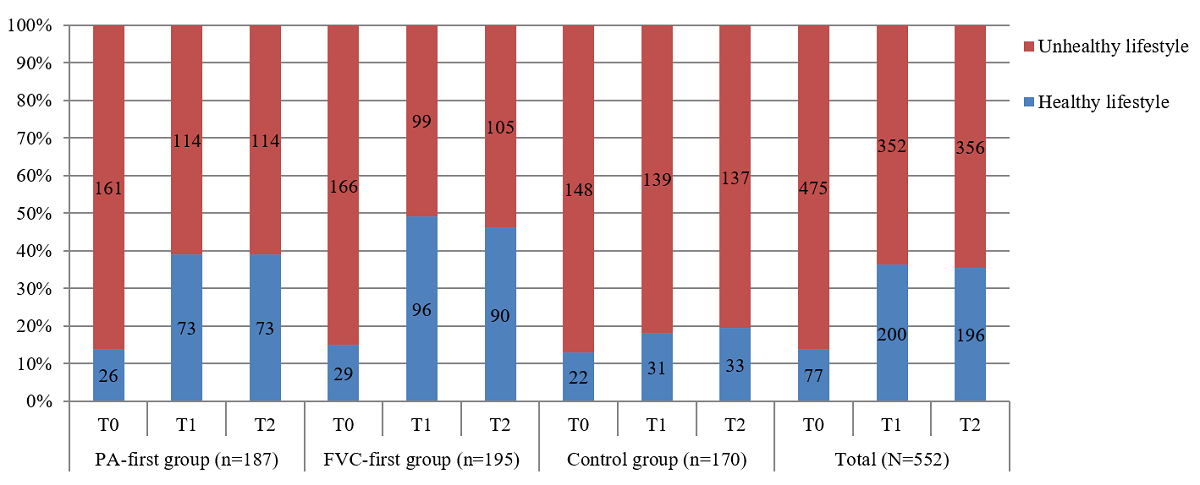
Descriptive information of lifestyle indicator from baseline assessment (T0) to follow-up assessment 12 weeks after baseline assessment (T2). FVC: fruit and vegetable consumption; PA: physical activity; T1: postintervention assessment 8 weeks after baseline assessment.

After the 8-week intervention (T1), both intervention groups, particularly the PA-first group, outperformed the control group in adhering to both PA and FVC behavioral recommendations (39% vs 18.2%). At the 1-month follow-up test (T2), the favorable effects were sustained for both intervention groups: 39% (73/187) of the participants in the PA-first group and 46.2% (90/195) of those in the FVC-first group adopted a healthy lifestyle, whereas only 19.4% (33/170) of the participants in the control condition did so (Figure 1).

To further explore the extent to which the intervention predicted the adoption of a healthy lifestyle at T1 and T2, binary logistic regression analyses were used (Table 2). First, all sociodemographic variables and intervention groups were used as predictors for the adoption of a healthy lifestyle at baseline. Neither of these variables showed a significant correlation to the lifestyle indicator (all *P*=.23-.49). When controlling for all sociodemographic variables and baseline lifestyle, the treatment was found to be a significant predictor for adopting a healthy lifestyle at both T1 and T2 (all *P*<.001). Specifically, after 8 weeks, participants in the PA-first and FVC-first groups were approximately 3.2 times and 5 times more likely, respectively, to practice or maintain a healthy lifestyle than those in the control group. After 12 weeks, participants receiving the interventions were approximately 3 times (PA first) and 4.1 times (FVC first) more likely to comply with a healthy lifestyle than the control group. The entire model accounted for 22% and 21% of the variance of the lifestyle indicator at T1 and T2, respectively.

**Table 2:** Intervention effects on the adoption of healthy lifestyles after 8 and 12 weeks (N=552).

Variable	Lifestyle^a^ at baseline (T0)	Lifestyle^a^ after 8 weeks (T1)	Lifestyle^a^ after 12 weeks (T2)
Constant	<.001	0.30	0.10
Lifestyle^a^ at baseline, OR^b^ (95% CI)	N/A^c^	0.14 (0.08-0.25)^d^	0.13 (0.07-0.23)^d^
PA^e^-first group, OR (95% CI)	1.10 (0.60-2.03)	3.24 (1.92-5.47)^d^	2.99 (1.78-5.03)^d^
FVC^f^-first group, OR (95% CI)	1.19 (0.65-2.17)	5.00 (2.98-8.40)^d^	4.07 (2.44-6.80)^d^
*R*	0.02	0.22	0.21

^a^0=adopted an unhealthy lifestyle (as reference); 1=adopted a healthy lifestyle.

^b^OR: odds ratio, adjusted for all sociodemographic variables.

^c^N/A: not applicable.

^d^*P*<.001.

^e^PA: physical activity (control group was set as reference).

^f^FVC: fruit and vegetable consumption (control group was set as reference).

### Intervention Effects on Psychosocial Determinants of Behavior Change

**Figure 2:**
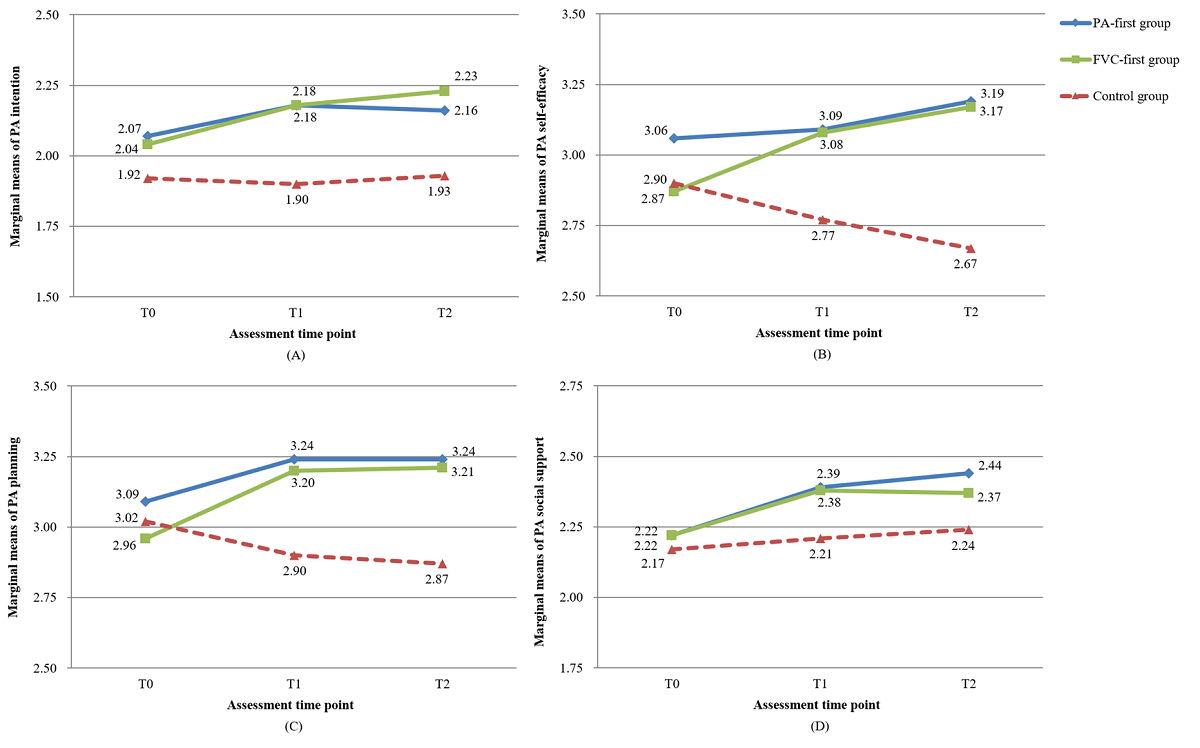
Marginal mean values of psychosocial determinants of physical activity (PA) change for 3 groups from baseline assessment (T0) to follow-up assessment 12 weeks after baseline assessment (T2). (A) Intention for PA. (B) Self-efficacy for PA. (C) Planning for PA. (D) Social support for PA. FVC: fruit and vegetable consumption; T1: postintervention assessment 8 weeks after baseline assessment.

**Figure 3:**
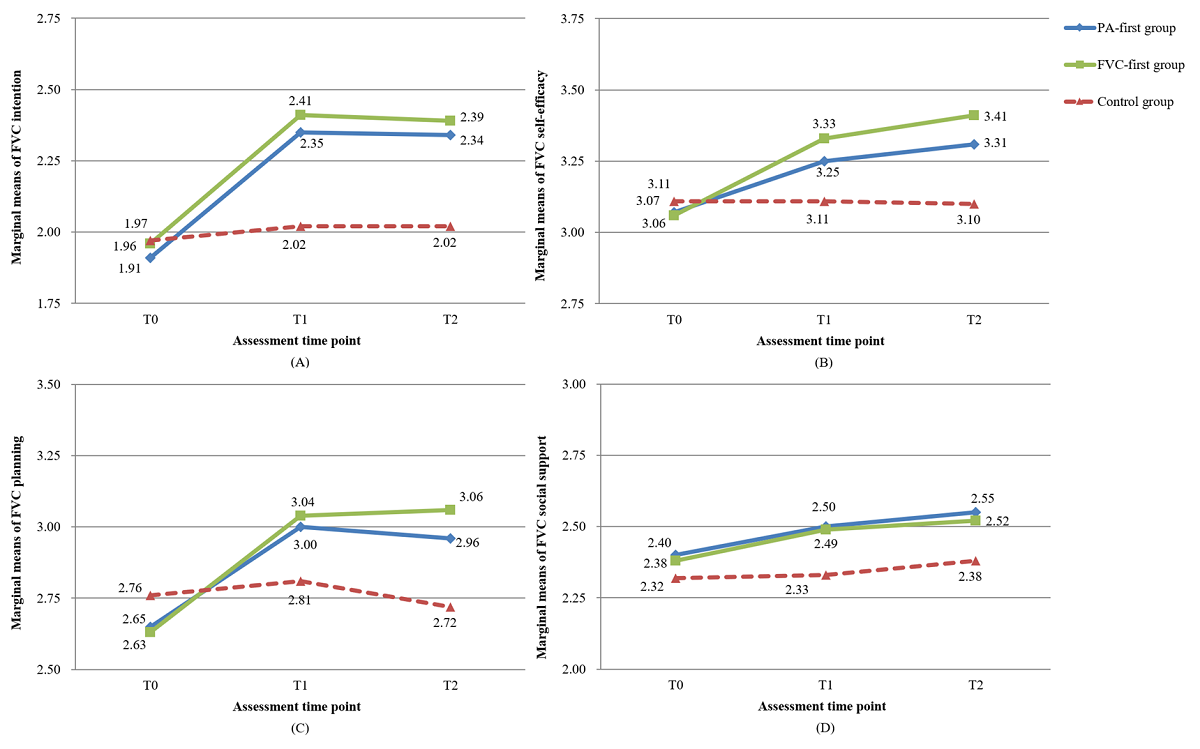
Marginal mean values of psychosocial determinants of fruit and vegetable consumption (FVC) change for 3 groups from baseline assessment (T0) to follow-up assessment 12 weeks after baseline assessment (T2). (A) Intention for FVC. (B) Self-efficacy for FVC. (C) Planning for FVC. (D) Social support for FVC. PA: physical activity; T1: postintervention assessment 8 weeks after baseline assessment.

The results of the linear mixed models showed that of the 8 *time* and *group* interactions, 4 (50%) were statistically significant (Tables 3 and 4). The marginal mean values of the psychosocial determinants of PA and FVC at 3 time points are presented in Figures 2 and 3.

Regarding the psychosocial determinants of PA, the interaction effect of time and treatment on self-efficacy (*P*<.001) and planning (*P*=.008) was significant for both intervention groups compared with the control group. After 8 weeks (T1), a significant between-group difference was found on intention (*P*<.001), self-efficacy (*P*=.01), and planning (*P*=.003-.008), with small-to-medium effect sizes (Cohen *d*=0.26-0.39), which was in favor of the 2 intervention groups. After 12 weeks (T2), the 2 intervention groups showed superiority in the improvement in all psychosocial determinants of PA (Cohen *d*=0.23-0.45), except for a nonsignificant difference in the perceived social support between the FVC-first and control groups (*P*=.16). Two intervention groups did not show any significant differences in the post hoc comparison (*P*=.43 at T1 and *P*=.93 at T2).

For psychosocial determinants of FVC, a statistically significant interaction effect was found on intention (*P*<.001) and planning (*P*<.001), whereas the time×group effect was marginally significant for self-efficacy (*P*=.06) and nonsignificant for social support (*P*=.83). After 8 weeks (T1), a significant between-group difference was found only in FVC intention (*P*<.001; Cohen *d*=0.39-0.45), which favored the 2 intervention groups. There were no significant between-group differences in other variables (*P*=.07-.67). After 12 weeks (T2), the 2 intervention groups showed a prominently higher level of intention (*P*<.001) and planning for FVC (*P*=.002-.04) than the control group. In addition, a significant difference in FVC self-efficacy was found between the FVC-first and control groups (*P*=.02), whereas the PA-first group showed a favorable change in FVC social support compared with the control group (*P*=.046).

**Table 3:** Results of the generalized linear mixed models with psychosocial mediators of physical activity (PA) change after 8 and 12 weeks as outcome measures (N=552).

Time and group		PA intention		PA self-efficacy			PA planning			PA social support	
		Value	Effect size, Cohen *d*	Value	Effect size, Cohen *d*	Value		Effect size, Cohen *d*	Value		Effect size, Cohen *d*
**Type III tests, *F*^a^**											
	Time×group	2.189	N/A^b^	5.55^c^	N/A	3.49^d^		N/A	0.83		N/A
	Time	5.88^d^	N/A	1.10	N/A	1.96		N/A	6.76^d^		N/A
	Group	5.49^d^	N/A	5.34^d^	N/A	3.73^e^		N/A	1.93		N/A
**After 8 weeks (T1), difference of marginal means^f^**											
	PA-first group versus control	0.28^c^	0.39	0.31^e^	0.27	0.34^d^		0.32	0.18		0.20
	FVC^g^-first group versus control	0.27^c^	0.38	0.30^e^	0.26	0.30^d^		0.30	0.17		0.19
	PA-first group versus FVC-first group	0.003	0.004	0.01	0.01	0.04		0.04	0.01		0.01
**After 12 weeks (T2), difference of marginal means^f^**											
	PA-first group versus control	0.30^d^	0.33	0.50^c^	0.43	0.37^d^		0.34	0.21^e^		0.23
	FVC-first group versus control	0.24^e^	0.26	0.52^d^	0.45	0.34^d^		0.31	0.13		0.15
	PA-first group versus FVC-first group	0.06	0.07	–0.02	–0.02	0.03		0.03	0.07		0.08

^a^Adjusted for baseline physical activity (metabolic equivalent minutes per week).

^b^N/A: not applicable.

^c^*P*<.001.

^d^*P*<.01.

^e^*P*<.05.

^f^Post hoc test: least significant difference.

^g^FVC: fruit and vegetable consumption.

**Table 4:** Results of the generalized linear mixed models with psychosocial mediators of fruit and vegetable consumption (FVC) change after 8 and 12 weeks as outcome measures (N=552).

Time and group		FVC intention			FVC self-efficacy			FVC planning			FVC social support	
		Value	Effect size, Cohen *d*	Value		Effect size, Cohen *d*	Value		Effect size, Cohen *d*	Value		Effect size, Cohen *d*
**Type III tests, *F*^a^**												
	Time×group	6.66^b^	N/A^c^	2.28		N/A	5.41^b^		N/A	0.37		N/A
	Time	39.06^b^	N/A	6.98^d^		N/A	17.62^b^		N/A	5.73^d^		N/A
	Group	5.90^d^	N/A	0.94		N/A	0.96		N/A	2.09		N/A
**After 8 weeks (T1), mean difference^e^**												
	PA^f^-first group versus control	0.33^b^	0.39	0.14		0.11	0.19		0.16	0.17		0.20
	FVC-first group versus control	0.39^b^	0.45	0.22		0.18	0.23		0.20	0.17		0.19
	PA-first group versus FVC-first group	–0.06	–0.07	–0.08		–0.07	–0.05		–0.04	0.01		0.01
**After 12 weeks (T2), mean difference^e^**												
	PA-first group versus control	0.32^b^	0.35	0.22		0.17	0.24^g^		0.22	0.18^g^		0.21
	FVC-first group versus control	0.37^b^	0.41	0.32^g^		0.25	0.35^e^		0.32	0.15		0.17
	PA-first group versus FVC-first group	–0.05	–0.06	–0.10		–0.08	–0.11		–0.10	0.03		0.04

^a^Adjusted for baseline fruit and vegetable consumption (portions per day).

^b^*P*<.001.

^c^N/A: not applicable.

^d^*P*<.01.

^e^Post hoc test: least significant difference.

^f^PA: physical activity.

^g^*P*<.05.

### Mediation Mechanisms of Immediate and Sustained Lifestyle Changes

Multicollinearity diagnostics revealed that there were no severe collinearity problems among the included psychosocial determinants of PA and FVC (correlation *r*=0.33-0.59, tolerance=0.45-0.72, variance inflation factor=1.40-2.25, eigenvalue=0.30-2.67, and condition index=1.00-2.93). Residualized change scores were obtained from the linear or binary regression analyses of T1 scores on T0 scores (ie, immediate change after 8 weeks) and of T2 scores on T0 scores (ie, sustained change after 12 weeks). All sociodemographic variables were included as covariates in the mediation analyses.

**Figure 4:**
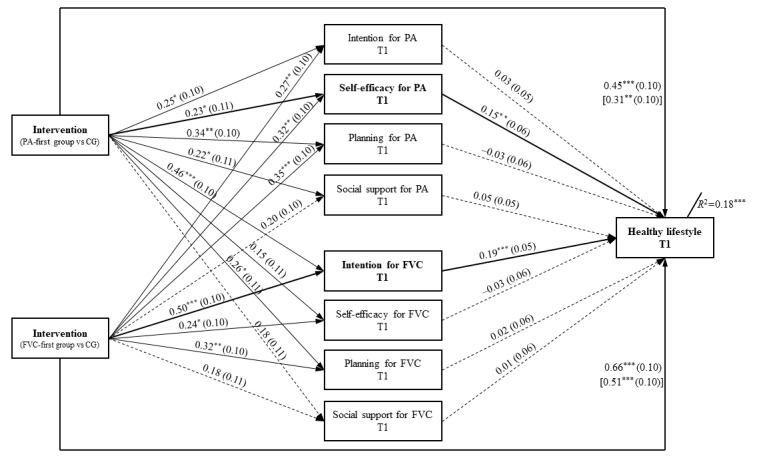
Mediation model of intervention effects on immediate lifestyle change at T1 (postintervention assessment 8 weeks after baseline assessment). CG: control group; FVC: fruit and vegetable consumption; PA: physical activity. *P<.05, **P<.01, ***P<.001.

After 8 weeks (T1), both intervention assignments significantly predicted the lifestyle changes (*b*_PA first_=0.45, 95% CI 0.25-0.65; *P*<.001; *b*_FVC first_=0.66, 95% CI 0.21-0.62; *P*<.001) and changes in all psychosocial determinants of behavior change, except social support (Figure 4). After controlling for the changes in psychosocial determinants, the associations between group assignments and lifestyle changes were attenuated but still statistically significant (*b*_PA first_=0.31, 95% CI 0.12-0.51; *P*=.002; *b*_FVC first_=0.51, 95% CI 0.31-0.70; *P*<.001), indicating that PA self-efficacy and FVC intention were partial mediators of intervention effectiveness. The multiple mediator model accounted for 17.5% of the variance in immediate lifestyle changes (*P*<.001).

**Figure 5:**
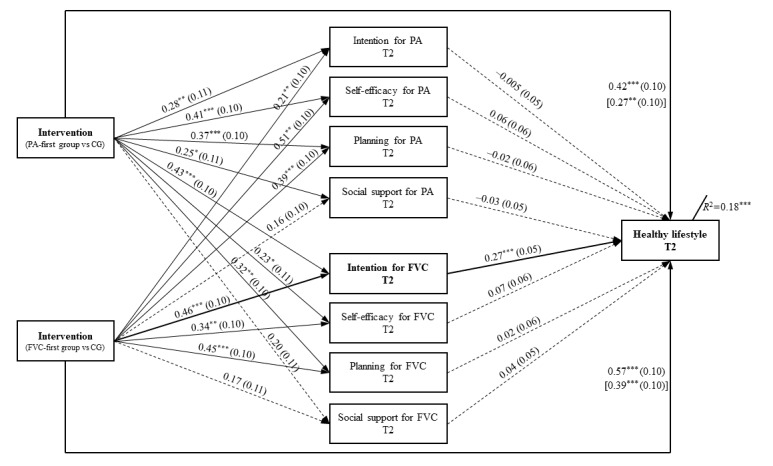
Mediation model of intervention effects on sustained lifestyle change at T2 (follow-up assessment 12 weeks after baseline assessment). CG: control group; FVC: fruit and vegetable consumption; PA: physical activity. *P<.05, **P<.01, ***P<.001.

After 12 weeks (T2), the intervention assignments continuously showed a significant prediction for lifestyle changes (*b*_PA first_=0.42, 95% CI 0.22-0.63; *P*<.001; *b*_FVC first_=0.57, 95% CI 0.37-0.78; *P*<.001) and changes in all psychosocial mediators, except social support (Figure 5). Among 8 mediators, only FVC intention was identified as a significant mediator that partially mediated the effects of both intervention groups on lifestyle changes at T2 (*b*=0.27, 95% CI 0.17-0.37; *P*<.001). The overall mediation model accounted for 18.4% of the variance in sustained lifestyle changes (*P*<.001).

## Discussion

### Principal Findings

This is one of the first studies to gain insights into web-based MHBC interventions for Chinese college students in terms of (1) the intervention effects on promoting a healthy lifestyle and enhancing psychosocial determinants of behavior change and (2) the mediation mechanisms of immediate and sustained lifestyle changes. The majority of the study hypotheses were supported.

The principal expected intervention effects on prompting a healthy lifestyle were identified (hypothesis 1). Compared with the participants in the placebo control condition, those in the intervention groups showed immediate and sustained improvements in adhering to both PA and FVC behavioral recommendations after receiving the 8-week web-based MHBC interventions, with medium effect sizes (*R*^2^=0.21-0.22). This finding is consistent with a previous computer-based intervention study with German employees [42,53], Iranian adolescents [20], and the general population in different countries [8,29]. Similar results were also presented in our previous study that aimed to enhance PA and FVC among outpatients with coronary heart disease during their home-based rehabilitation [43]. Taken together, our findings add evidence regarding the potential of web-based MHBC interventions for promoting a healthy lifestyle among young adult populations.

Regarding the intervention effects on the psychosocial determinants of behavior change, 50% (4/8) were found to be statistically significant. Research hypotheses 2a and 2b were partially supported. Although a favorable improvement was detected for the 2 intervention groups descriptively, we could not find a statistically significant time and treatment interaction on intention for PA, self-efficacy for FVC, and social support for both PA and FVC. The findings were inconsistent with those of previous studies of college students and other populations that had indicated a significant intervention effect on these variables [38]. One potential interpretation could be that the ceiling effect came into play here [54]. In particular, the participants in this study had a high level of intention for PA (mean 2.22, SD 0.71; scale scoring range 1-4) and high perceived social support for both health behaviors (mean_PA_ 2.23, SD 0.91; mean_FVC_ 2.37, SD 0.86) at baseline. In addition, our findings might be attributed to the impacts of external social and environmental factors (eg, university policy and environmental barriers). In our previous qualitative interviews, these participants had stated that their health behaviors are considerably affected by the mandatory university policy for PA in terms of the *Ham Run task* (ie, all undergraduates had to complete a 2000-meter run 28 times, accounting for 20% of the PE course credit) and barriers to FVC (eg, financial issues and limited provision of fruit and vegetables at university canteens) [34]. Unsurprisingly, in such a case these external sources might, to some extent, suppress the intervention effects on the internal sources of behavior change (eg, intention for PA and self-efficacy for FVC). As our research focused on individual-level psychosocial determinants of behavior change, the social and environmental factors were not involved. This should be systematically examined in future studies.

For mediation analyses (hypotheses 3a and 3b), only intention and self-efficacy were identified as salient mediators of lifestyle changes. In particular, compared with the control condition participants, those in the intervention groups who gained more self-efficacy for PA and who increased more intention for FVC were more likely to show a successful change in lifestyle after 8 weeks (ie, immediate change). This finding is consistent with that of previous studies of workplace employees and clinical patients [42,43,53], demonstrating the importance of empowering the internal sources (intention and self-efficacy) in facilitating both sufficient PA and healthy diets. For the sustained lifestyle change (after 12 weeks), only intention for FVC was identified as a significant mediator of intervention effectiveness. This finding supplements the evidence for emphasizing the role of intention in maintaining long-term change of lifestyle behaviors [55,56]. The hypothesized role of planning and social support in facilitating a healthy lifestyle has not been found in our study, and further investigation is warranted. In addition, the mediation models only showed medium effect sizes in explaining the variance of lifestyle changes among Chinese college students (*R*^2^=0.18), which are comparatively higher than those of German workplace employees (*R*^2^=0.10) [42] and lower than those of Chinese outpatients with coronary heart disease (*R*^2^=0.33) [43]. Further studies with inclusion of more psychosocial mediators are warranted.

### Limitations

Several limitations should be noted. First, the behavioral indicators were evaluated using self-reported measures, which may lead to recall bias and social desirability effect [57]. The inclusion of objective measures such as accelerometers, pedometers, and digital cameras, which can provide more accurate and reliable assessments of health behaviors, is recommended in future studies. Second, the RCT design was used in consideration of the feasibility and limited resources for study implementation; however, this may lead to spillover effect and contamination [58]. Although we applied several strategies to minimize this problem and did not identify any contamination in our previous study, a stricter design (eg, cluster RCT) should be used, if possible. In addition, the intervention effects may be confounded by external sources (eg, season, university policy, PA facilities, and environmental barriers) [59]. Further investigation considering these factors is warranted. In addition, following a parsimonious principle and considering the characteristics of the study sample, we did not include habit strength and action control as in our previous intervention program. Accordingly, the role of these factors in facilitating a healthy lifestyle has not been examined in this study. However, further identification of the mediating effect of these factors is needed [60]. In addition, because this is a secondary analysis of our previous RCT targeting specific outcomes (healthy lifestyle as well as psychosocial determinants of PA and FVC), the findings generated in this study cannot be regarded as representative of all student samples who receive the web-based MHBC intervention, and caution is needed when generalizing to wider populations. Finally, our study focused on the lifestyle pattern combining only PA and FVC; more lifestyle behaviors (eg, sedentary behaviors, sleep patterns, smoking, and alcohol addiction) are deserving of inclusion in future studies to contribute to a better understanding of comprehensive lifestyle patterns. Despite these limitations, this study may have considerable implications for future MHBC research and practice on promoting a healthy lifestyle among college students in terms of addressing PA self-efficacy and FVC intention. Our findings supplement evidence on the effectiveness of web-based MHBC interventions independently of whether PA or FVC is targeted first. The study adds new knowledge about the underlying mechanisms of successful MHBC interventions in terms of lifestyle approaches that require combined strategies.

### Conclusions

To conclude, this study demonstrated the great potential of 8-week theory-based and web-based MHBC interventions for promoting a healthy lifestyle and several psychosocial determinants of behavior change among Chinese college students. This study also identified a salient mediating effect of intention and self-efficacy in facilitating successful, immediate, or sustained lifestyle changes. The research findings provide empirical evidence for future MHBC research and practice among young adult populations: lifestyle can be improved independently of whether PA or FVC is addressed first by means of web-based interventions. Further investigation on the effects in other populations and countries and with other behaviors, such as healthy internet use and stress management, is needed.

## Abbreviations

CONSORT: Consolidated Standards of Reporting Trials
FVC: fruit and vegetable consumption
HAPA: health action process approach
MHBC: multiple health behavior change
PA: physical activity
RCT: randomized controlled trial
VAS: visual analog scale
